# Applying Deep Learning for Breast Cancer Detection in Radiology

**DOI:** 10.3390/curroncol29110690

**Published:** 2022-11-16

**Authors:** Ella Mahoro, Moulay A. Akhloufi

**Affiliations:** Perception, Robotics and Intelligent Machines Research Group (PRIME), Department of Computer Science, Université de Moncton, Moncton, NB E1A 3E9, Canada

**Keywords:** breast cancer, deep learning, convolutional neural network, classification, detection, segmentation, radiology

## Abstract

Recent advances in deep learning have enhanced medical imaging research. Breast cancer is the most prevalent cancer among women, and many applications have been developed to improve its early detection. The purpose of this review is to examine how various deep learning methods can be applied to breast cancer screening workflows. We summarize deep learning methods, data availability and different screening methods for breast cancer including mammography, thermography, ultrasound and magnetic resonance imaging. In this review, we will explore deep learning in diagnostic breast imaging and describe the literature review. As a conclusion, we discuss some of the limitations and opportunities of integrating artificial intelligence into breast cancer clinical practice.

## 1. Introduction

Breast cancer is the most common cancer and can affect both women and men. An abnormal growth of breast cells leads to breast cancer, and as these cells continue to multiply at a faster rate than healthy cells, they accumulate and form a mass [1].

Mutations in two genes known as breast cancer (BRCA) genes, BRCA1 and BRCA2, are linked with 5 to 10% of all breast cancers [2]. There is an increased risk of breast cancer for those who inherit harmful variants in one of these genes. BRCA1 or BRCA2 variants can be inherited from a parent. If a parent carries any mutation in one of these genes, then the child has a 50% chance of inheriting the mutation [3]. It is possible for toxins, radiation and chemicals to harm these genes during adulthood [4].

Breast cancer is classified based on the cells in the breast that eventually become cancerous [5]. There are many types of breast cancer. The first type is ductal carcinoma in situ (DCIS), a non-invasive cancer present in the lining of the breast milk duct. The second type is invasive ductal carcinoma, which is the most common type and makes up about 70–80% of all breast cancers. The third type is inflammatory breast cancer, a form of invasive breast cancer in which cancer cells block lymph vessels, which causes the breast to look inflamed. It is rare and is responsible for about 1% to 5% of all breast cancers. The fourth type is triple-negative breast cancer, an aggressive form of invasive breast cancer in which the cancer cells lack estrogen receptors (ER) or progesterone receptors (PR) and do not produce human epidermal growth factor receptor 2 (HER2). About 15% of all breast cancers are of this type. Other types of breast cancer are less common and make up around 1% of all breast cancers [6].

There are several ways to detect breast cancer. Breast self-examination can be conducted by pressing on the breast and checking for changes [7]. However, this method is not very reliable in detecting cancer. In 1960, mammography was introduced [8]. It is one of the most common screening tools used to detect breast cancer [9]. This type of screening may be uncomfortable and painful for the patient due to the pressure applied to the breast to spread the breast tissue and enhance the clarity of the X-ray image [10]. In addition to mammography, other types of screening have been introduced, such as thermography, ultrasound, and magnetic resonance imaging (MRI) [11].

The early detection and treatment of breast cancer increases the survival rates [12]. Unfortunately, in most cases, breast cancer is detected after symptoms appear rather than through screening. Delaying treatment may result in the cancer reaching an advanced stage and decreasing the chances of survival [13]. A number of strategies have been developed to detect breast cancer in its early stages.

Artificial intelligence technology plays a major role in diagnosis and decision making in the medical field [14]. Many applications were developed using machine learning and deep learning to assist with a number of tasks, such as the classification, detection and segmentation of breast cancer. Currently, deep learning is the most popular method [15].

The novel aspect of this study relates to the review of the most recent research on the application of deep learning to multiple medical imaging modalities for breast cancer diagnosis, particularly the most common screening tests for breast cancer used in deep learning: mammography, thermography, ultrasound, and magnetic resonance imaging (MRI).

The main contributions of this review are as follows:

- Our paper provides a detailed review on the available breast cancer datasets of each of the 4 different modalities not available in past reviews [16,17,18].
- We explore the methods in which recent deep learning algorithms are used to detect breast cancer using different types of screening.
- We discuss some of the limitations and opportunities of integrating artificial intelligence into breast cancer screenings.

The structure of this review is as follows: Section 2 explains the fundamentals of deep learning in medical and cancer research, Section 3 describes the data availability for various types of breast cancer screening, and Section 4 provides a detailed review of breast cancer detection research using deep learning. The discussion follows in Section 5, and the conclusion is presented in Section 6.

## 2. Deep Learning Methods

The term deep learning refers to a sub-field of machine learning in which several processing layers are employed to extract data features relevant to a given task [19]. Using multiple layers of neural network architectures and a large amount of labeled data, models are trained and can make classifications based on images, texts or sounds [20].

To detect breast cancer, different deep learning tasks can be applied:

- Image classification is the probability that the input is a particular class [21]. It involves defining a set of target classes (e.g., cancerous, healthy) and using labeled images to train a model to recognize them. Raw pixel data are the input to early computer vision models.
- Object detection refers to locating and presenting the abnormal areas of an image, such as tumors [22]. A bounding box is drawn around one or more objects in an image to localize them.
- Image segmentation is the task of grouping parts of an image that reflect the same object class (e.g., tumor area). The process of determining the class of each pixel is made by combining classification and object detection [23].

**Figure 1:**
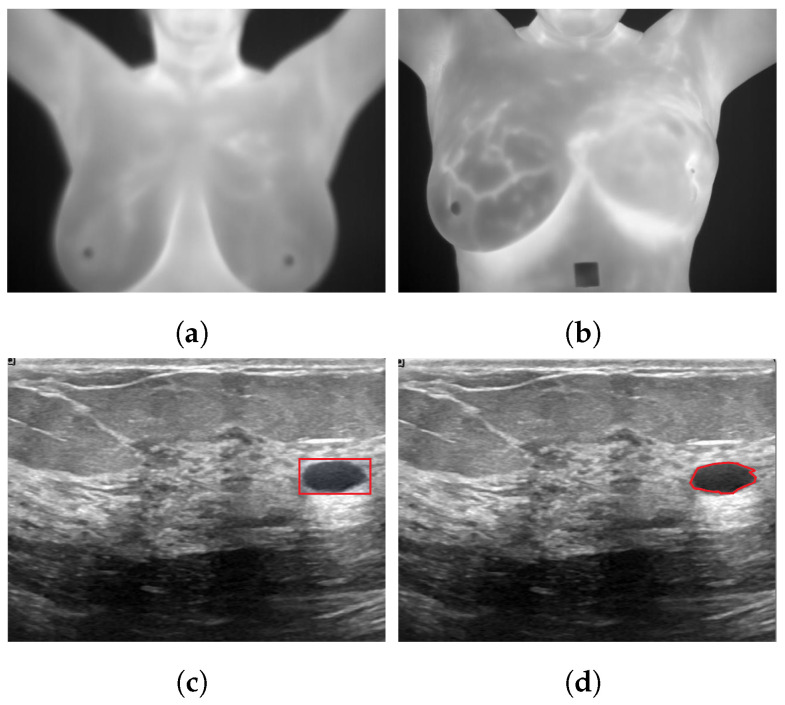
Sample applications of deep neural networks to breast cancer images for (**a**): classification as healthy using a thermogram; (**b**): classification as unhealthy using a thermogram [24]; (**c**): detection using ultrasound images; (**d**): segmentation using ultrasound images [25].

Figure 1 illustrates various deep learning tasks for breast cancer detection.

### 2.1. Layers

A layer is a basic structure of neural networks in which the information from the previous layers is passed to the next layer [26]. There are the most common layers such as convolution layers [27], pooling layers [28], dense layers [29], dropout layers [30] and batch normalization layers [31].

- A convolution layer employs a filter to scan the input with respect to its dimensions in order to extract features based on the input. There are 3 types of convolution layers: 1D conv, 2D conv and 3D conv. The filter size, stride and padding are its hyperparameters, and as a result, feature maps are produced.
- A pooling layer is typically employed after a convolution layer to extract features and reduce dimensions by downsampling. The most popular types of pooling used are max pooling, average pooling and global average. The padding and pool size are its hyperparameters.
- A dropout layer is, in general, used to prevent the model from overfitting. When the training phase is updated, the output of a subset of hidden units is randomly set to 0.
- A dense layer is a regular fully connected layer in which every neuron receives input from all neurons in its preceding layer. It is the most commonly used layer for classification.
- A normalization layer is used to standardize the input. Different types of normalization layers exist, such as batch normalization, weight normalization, layer normalization and group normalization.
- An activation layer carries out an activation function on the previous layer and increases the non-linearity of the network. There are several used activation functions, such as ReLU, Sigmoid, Tanh, Softmax, leaky ReLU, etc.

### 2.2. Loss Function

When a neural network model is trained to perform a particular task, such as regression or classification, the loss function measures the performance of the model [32]. It measures the difference between the predicted classes and ground truth classes. It plays an important role in the performance of the model. The following are the most common loss functions used in the reviewed papers.

#### 2.2.1. Cross-Entropy Loss

Cross-entropy loss is a loss function used to measure the efficiency of a classification model [33].

1. Binary cross-entropy loss
  (1) $$\begin{array}{c} l\left(y,\hat{y}\right)=-\left(y\log\left(\hat{y}\right)+\left(1-y\right)\log\left(1-\hat{y}\right)\right); \end{array}$$
2. Multi-class cross-entropy loss
  (2) $$\begin{array}{c} l\left(y,\hat{y}\right)=-\sum_{i=1}^{n}y_{i}\log\left({\hat{y}}_{i}\right). \end{array}$$

-
n=number of classes;

-
$y_{i}=ground\;truth\;classes;$

- ${\hat{y}}_{i}=probability\;of\;the\;predicted\;classes$.

#### 2.2.2. YOLO Loss

YOLO calculates loss based on the sum-squared error between predictions and ground truth. The loss function combines the loss of classification, localization and confidence. It is used for object detection [34].

(3) $$l1=\lambda_{coord}\sum_{i=0}^{s^{2}}\sum_{j=0}^{B}1_{ij}^{o}bj\left[\left(x_{i}-{\hat{x}}_{i}^{2}\right)+{\left(y_{i}-{\hat{y}}_{i}\right)}^{2}\right]+\lambda_{coord}\sum_{i=0}^{s^{2}}\sum_{j=0}^{B}1_{ij}^{o}bj\left[\left(\sqrt{w_{i}}-{\sqrt{\hat{w_{i}}}}^{2}\right)+{\left(\sqrt{h_{i}}-\sqrt{\hat{h_{i}}}\right)}^{2}\right]\;\begin{array}{c} +\sum_{i=0}^{s^{2}}\sum_{j=0}^{B}1_{ij}^{o}bj\left[{\left(p_{i}\left(n\right)-{\hat{p}}_{i}\left(n\right)\right)}^{2}\right]+\sum_{i=0}^{s^{2}}\sum_{j=0}^{B}1_{ij}^{o}bj\left[\left(C_{i}-{\hat{C}}_{i}^{2}\right)\right]+\lambda_{coord}\sum_{i=0}^{s^{2}}\sum_{j=0}^{B}1_{ij}^{o}bj\left[\left(C_{i}-{\hat{C}}_{i}^{2}\right)\right] \end{array}$$

-
λ=weighted parameter for balancing;

-
n=classes;

-
$x_{i},y_{i}=bbox’s\;center\;coordinate\;ground\;truth;$

-
${\hat{x}}_{i},{\hat{y}}_{i}=bbox’s\;center\;coordinate\;prediction;$

-
B=number of bbox;

-
$w_{i},h_{i}=bbox’s\;width\;and\;height\;ground\;truth;$

-
${\hat{w}}_{i},{\hat{h}}_{i}=bbox’s\;width\;and\;height\;prediction;$

-
$p_{i}=class\;prediction;$

-
${\hat{p}}_{i}=class\;ground\;truth;$

-
s=number of cells in bbox;

-
$C_{i}=confidence\;score\;of\;if\;there\;is\;an\;object;$

-
${\hat{C}}_{i}=is\;1\;if\;there\;is\;an\;object,\;else\;0.$

#### 2.2.3. IoU Loss

The intersection over union (IoU) loss function is a scale-invariant bounding box regression function. It is used for object detection [35].

(4) $$\begin{array}{c} L_{\mathrm{IoU}}=1-\frac{|B\cap B_{gt}|}{|B\cup B_{gt}|} \end{array}$$

-
B=predicted bounding box;

-
$B_{gt}=ground\;truth\;bounding\;box.$

#### 2.2.4. GIoU Loss

The generalized intersection over union (GIoU) loss function has a penalty term along with the IoU loss function and is used for detection [36].

(5) $$\begin{array}{c} L_{\mathrm{GIoU}}=1-IoU+\frac{|C-B\cup B_{gt}|}{\left|C\right|} \end{array}$$

- IoU = $\frac{|B\cap B_{gt}|}{|B\cup B_{gt}|};$
- *C* is the smallest box covering *B*;
- B= predicted bounding box;
- $B_{gt}=$ ground truth bounding box.

#### 2.2.5. Smooth L1 Loss

Smooth L1 loss is a loss function for object detection that makes bounding box regression more robust [37].

(6) $$\begin{array}{c} \mathrm{smooth}\;L1=\left\{\begin{array}{cc} 0.5f^{2} & |f|\leq 1\\ |f|-0.5 & \mathrm{otherwise} \end{array}\right. \end{array}$$

f is the difference between the predicted value and the ground truth.

#### 2.2.6. Focal Loss

Focal loss is a RetinaNet loss function used for object detection that helps to handle the class imbalance problem during training [38].

$$FL\left(p_{t}\right)=-{\left(1-p_{t}\right)}^{\gamma }log\left(p_{t}\right)$$

-
$p_{t}=\left\{\begin{array}{cc} p & \mathrm{if}\;y\;=\;1\;\\ \left(1-p\right) & \mathrm{otherwise}; \end{array}\right.$

- p is the probability that the model estimated for the class with the label *y* = 1;
- *y*= ground truth class;
- γ controls the shape of the curve.

#### 2.2.7. Dice Loss

Dice loss is a widely-used loss to calculate the similarity between images and is used for segmentation [39].

(7) $$\begin{array}{c} DL\left(y,\hat{y}\right)=1-\frac{2y\hat{y}+1}{y+\hat{y}+1} \end{array}$$

- y= ground truth classes;
- $\hat{y}=$ probability of the predicted classes.

#### 2.2.8. Shape-Aware Loss

Shape-aware loss is a loss function in which shape is taken into account [40]. It is used for segmentation.

(8) $$\begin{array}{c} L_{shape-aware}=-\sum_{i}CE\left(y,\hat{y}\right)-\sum iE_{i}CE\left(y,\hat{y}\right) \end{array}$$

-
$CE=\left\{\begin{array}{cc} -\log(\hat{y}) & \mathrm{if}\;y\;=\;1\;\\ -\log(1-\hat{y}) & \mathrm{otherwise} \end{array}\right.$

- y= ground truth classes;
- $\hat{y}=$ probability of the predicted classes;
-
$E_{i}=D\left(\hat{C},C_{GT}\right);$

- D= curve of the Euclidean distance;
- $\hat{C}=$ curve of the predicted segmentation;
- $C_{GT}=$ curve of the ground truth.

### 2.3. Metrics

In the reviewed papers, the common performance metrics in breast cancer detection are accuracy, sensitivity, specificity, precision, F1-score, receiver operating characteristic (ROC) curve and area under the curve (AUC) [41]. The true positive (TP) represents the number of positive classes that have been correctly classified as positive. The true negative (TN) is the number of negative classes that that have been correctly classified as negative. The false positive (FP) represents the number of negative classes that have been misclassified as the positive class. The false negative (FN) represents the number of positive classes that have been misclassified as negative.

Table 1 summarizes the common performance metrics for various deep learning tasks in the reviewed papers.

## 3. Public Datasets

There are many datasets available for breast cancer. Many of them are publicly available.

### 3.1. Mammographic Image Analysis Society Digital Mammogram Database (MIAS)

**Figure 2:**
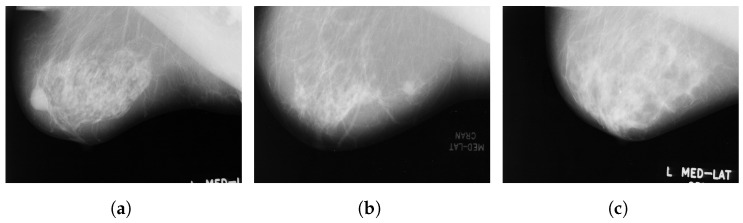
Sample images from the MIAS database (**a**): benign; (**b**): malignant; (**c**): normal.

The Mammographic Image Analysis Society Digital Mammogram Database (MIAS) [42] consists of 322 images collected in 1994 from 161 patients and stored in portable gray map file format (PGM) with a spatial resolution of 8 bits per pixel. This database consists of three classes: benign, malignant and normal. Figure 2 illustrates some sample images from the MIAS database.

### 3.2. Magic-5

Magic-5 [43] is an Italian database of digitised mammograms that was collected in 1999 by a group of physicists. As a first step, they worked with radiologists in several Italian hospitals to develop a computer-aided detection system. The database contains 3369 images collected from 967 patients and classified according to lesion type, morphology, breast tissue and pathology type with a resolution of 16 bits per pixel and saved in a DICOM format. There are more than 60% of patients in the database who are older than 50 years of age. However, these images were collected in diverse environments and are from multiple views, including lateral, craniocaudal (CC) and mediolateral oblique (MLO) views. The repartition of the database in the CC, MLO and lateral views is 1601, 1456 and 312 images, respectively.

### 3.3. The BancoWeb LAPIMO Database

The BancoWeb LAPIMO Database [44] contains 1473 images collected in 2010 from 320 cases. Images are available in TIFF format in 12-bit resolution. In general, their spatial resolutions are between 0.075 mm and 0.150 mm, depending on the scanner used. There are three classes of data in this database, namely benign, malignant and normal. All images are attached to the medical reports of the corresponding exams. In most cases, the exams were performed for women between the ages of 40 and 60. Access is available for registered users to Breast Imaging-Reporting And Data System (BI-RADS) annotations and patient information.

### 3.4. INbreast

**Figure 3:**
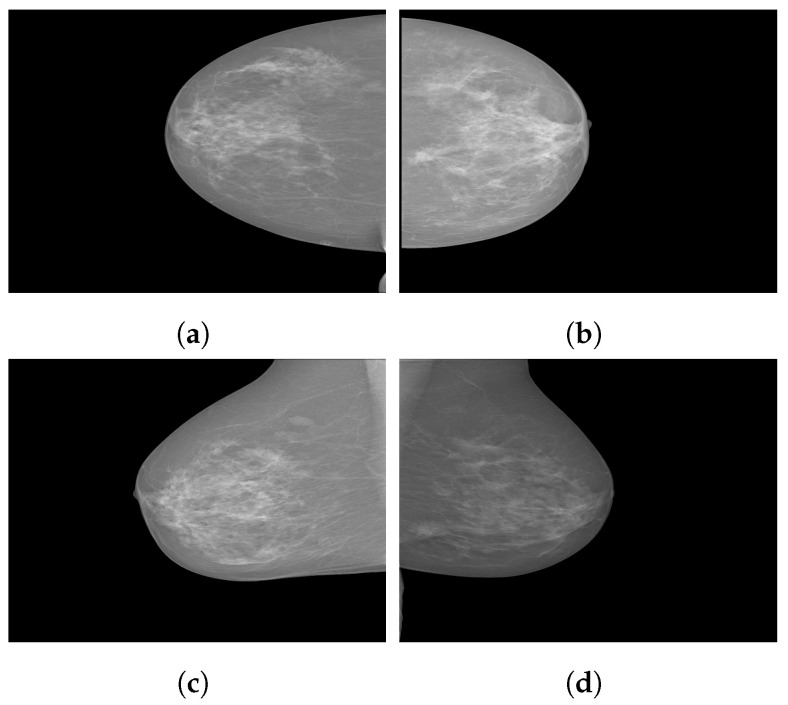
Sample images from the INbreast database. (**a**) Craniocaudal (CC) view of the right breast; (**b**) CC view of the left breast; (**c**) mediolateral oblique (MLO) view of the right breast; (**d**) MLO view of the left breast.

INbreast [45] is a full-field digital mammographic database that consists of 410 images collected in 2012 at a breast center located in a university hospital (CHSJ, Breast Centre, Porto). Images were taken from screening, diagnostic and follow-up cases. From 115 cases, 90 have two images from craniocaudal (CC) and mediolateral oblique (MLO) views of each breast, and there are 25 cases where two images of only one breast were collected. Among the 91 cases with 2 images per breast, 8 had acquired images at various times. The dataset is divided into six classes: asymmetries, calcifications, masses, multiple findings and architectural distortions. These images are available in a DICOM format with a resolution of 14 bits per pixel. The region of interest of each image and related information are stored in XML format. Figure 3 illustrates some sample images from the INbreast database.

### 3.5. Digital Database for Screening Mammography (DDSM)

The Digital Database for Screening Mammography (DDSM) [46] was first collected in 1999 from Massachusetts General Hospital, the Wake Forest University School of Medicine, Sacred Heart Hospital and Washington University of the St. Louis School of Medicine. This database contains 2620 film mammography cases with 4 standard views (mediolateral oblique and craniocaudal) from each case. A total of 10,480 images are divided into 3 classes: normal, benign and malignant. The DDSM is widely used due to its large database and ground truth validation. The images are available in LJEPG format with a resolution of 8 or 16 bits per pixel.

### 3.6. CBIS-DDSM

The CBIS-DDSM (Curated Breast Imaging Subset of the DDSM) [47] is an updated and standardized version of the DDSM set up in 2017 that contains a subset of the DDSM data that has been selected and curated by trained radiographers and converted into DICOM format. It consists of 1644 cases with a spatial resolution of 16 bits per pixel, along with updated ROI segmentation and bounding boxes, as well as pathologic diagnosis for training data.

### 3.7. OPTIMAM Medical Image Database

The OPTIMAM Mammography Image Database (OMI-DB) [48] has been collected since 2010 to provide a centralized, fully annotated dataset for research. The Cancer Research United Kingdom created the database to evaluate how various factors affect mammograms for breast cancer detection and collected 2,889,312 images from 173,319 women. The 173,319 women include 154,832, 6909, 9690 and 1888 with normal breasts, benign findings, screen-detected cancers, and interval cancers, respectively [17]. These images are available in DICOM format. Additionally, the OMI-DB includes clinical information about previous analog imaging sessions, but images are not included.

### 3.8. Breast Cancer Digital Repository (BCDR)

The Breast Cancer Digital Repository (BCDR) [49] is a collection of patient data in northern Portugal collected since 2009 that has been validated by radiologists at Hospital São João, University of Porto, Portugal. It is composed of 1010 cases, including 3703 digitized film mammography images available in TIFF format with a resolution of 14 bits per pixel, clinical history, segmented lesions that are BI-RADS classified, and image-based descriptors.

Additionally, manual segmentation of identified lesions has been made by medical specialists. There were 795 lesions segmented in MLO and CC images, yielding a total of 1493 segmentations, in which 639, 341, 145, 102, 66 and 2 are masses, microcalcifications, calcifications, stromal distortions, architectural distortions and axillary adenopathies, respectively.

### 3.9. DMR-Database for Mastology Research-Visual Lab

**Figure 4:**
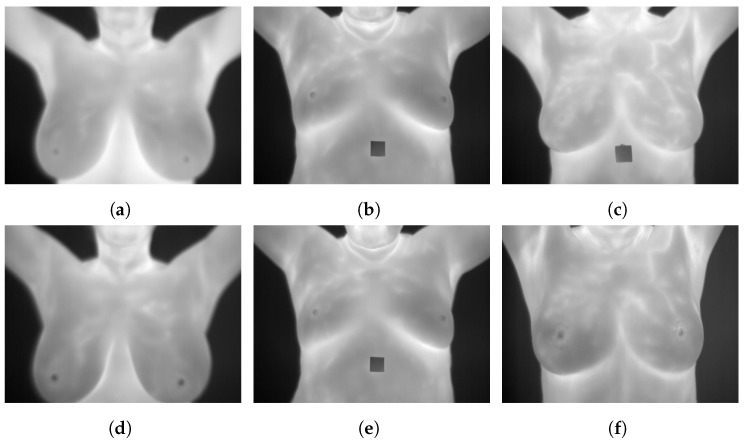
Sample images from the DMR-Database (**a**): static frontal healthy; (**b**): static frontal sick; (**c**): static frontal unknown; (**d**): dynamic frontal healthy; (**e**): dynamic frontal sick; (**f**): dynamic frontal unknown.

The DMR-Database [24] has been collected since 2010 by the Database for Mastology Research-Visual Lab, UFF, Niteroi, Brazil. This dataset was taken by FLIR SC-620 thermal cameras with 480 × 640 pixel resolution. Static and dynamic protocols were used to acquire the images. Static images are recorded after 10 to 15 min of thermal stabilization while the patient rested. However, the dynamic images are comprised of a sequence of thermograms taken every 15 s during five minutes. Static protocols reduce the formation of false regions of warmth or cold by uniformly cooling the skin of the breasts and armpits. However, there is a long resting time needed to stabilize skin temperature. A total of 5760 images are divided into 3 classes, healthy, sick, and unknown, and are available in JPG format. Figure 4 illustrates some sample static and dynamic images from the DMR-Database.

### 3.10. Breast Ultrasound Image

**Figure 5:**
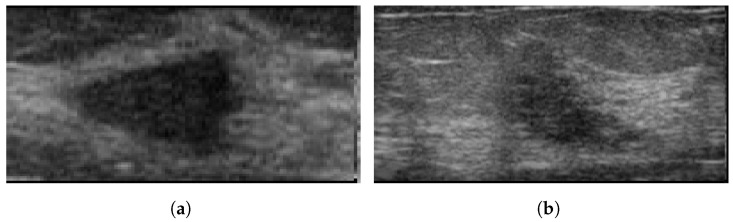
Sample images from the BUS database. (**a**): benign; (**b**): malignant.

Breast Ultrasound Image (BUS) [50], collected in 2017, is a publicly available Mendeley dataset. The dataset consists of 250 breast cancer images, of which 100 are benign and 150 are malignant. The size of the images are 72 × 72 pixels, ranging in width from 57 to 61 pixels and height from 75 to 199 pixels [51]. These images are available in BMP format. It has been widely used in various studies [52,53,54]. Figure 5 illustrates some sample images from the Breast Ultrasound Image (BUS) dataset.

### 3.11. Breast Ultrasound Dataset

**Figure 6:**
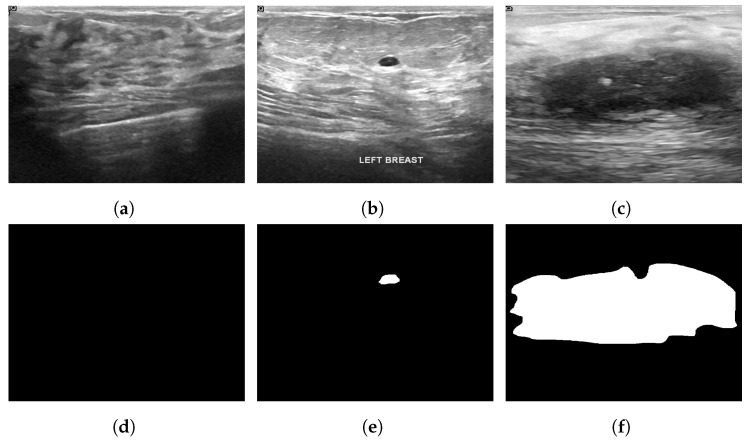
Sample images from the Breast Ultrasound dataset (**a**): normal; (**b**): benign; (**c**): malignant; (**d**): ground truth—normal; (**e**): ground truth—benign; (**f**): ground truth—malignant.

The Breast Ultrasound Dataset [25] was collected in 2018 and stored in DICOM format at Baheya Hospital, Cairo, Egypt. A LOGIQ E9 ultrasound system and a LOGIQ E9 Agile ultrasound system were used in the scanning process. Using a DICOM converter application, the images were converted into PNG format. All images were preprocessed by removing duplicate images and unused boundaries from the images. A total of 600 female patients between the ages of 25 and 75 were included in this dataset. The dataset was divided into three classes: normal, benign and malignant, with 133, 487 and 210 images, respectively. Each image is in PNG format and is 500 pixels by 500 pixels. There is also a ground truth available for each image. Figure 6 illustrates some sample images from the Breast Ultrasound dataset.

### 3.12. Dynamic Contrast-Enhanced Magnetic Resonance Images

Dynamic contrast-enhanced magnetic resonance images of breast cancer patients with tumor locations (Duke-Breast-Cancer-MRI) [55] is a dataset collected from 2000 to 2014 and stored in the National Cancer Institute’s Cancer Imaging Archive. The dataset includes 922 DCE-MRI images of invasive breast cancer patients before treatment, along with information about demographics and clinical, pathological and treatment outcomes. The dataset is available in a DICOM format and was acquired by 1.5 T or 3 T scanners in the prone position. It consisted of a non-fat-saturated T1-weighted sequence, a fat-saturated gradient echo T1-weighted precontrast sequence, and mostly three to four post-contrast sequences. Annotation boxes were provided by radiologists to locate the tumors on the DCE-MRI images.

### 3.13. RIDER Breast MRI

The Reference Image Database to Evaluate Therapy Response (RIDER) Breast MRI [56] was collected in 2006 to establish procedures to evaluate the efficacy of drugs or radiation treatments. The dataset is stored in the National Cancer Institute’s Cancer Imaging Archive and is composed of 1500 images available in DICOM format obtained from an approach that identifies the changes in the local apparent diffusion coefficient in 3 of 5 primary breast cancer patients.

**Figure 7:**
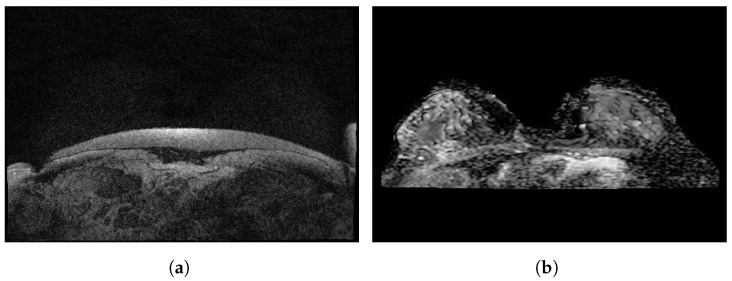
Sample images from (**a**): Duke-Breast-Cancer-MRI database; (**b**): RIDER Breast MRI database.

Figure 7 illustrates some sample images from the Duke-Breast-Cancer-MRI and RIDER Breast MRI databases.

Table 2 summarizes the characteristics of the reviewed public datasets.

## 4. Deep Learning for Breast Cancer

A breast cancer screening involves examining breasts using imaging modalities to see if there are any unusual signs or symptoms of cancer, which can help detect the disease at an early stage. In the following, we present deep learning research work conducted using different types of screening for breast cancer detection.

### 4.1. Mammography

Mammography is a type of breast cancer screening used to detect cancer at an early stage with a low dose of radiation. There are three types of mammograms [57]. The first type is film mammography, which is an X-ray image of the breast. The second type is digital mammography, which is a computer image of the breast. The third type is digital breast tomosynthesis (DBT), which involves taking a series of pictures of the breast from various angles. Many studies have been conducted using mammograms to detect breast cancer.

Using a fully annotated dataset, Shen et al. [58] proposed a deep learning algorithm with an end-to-end training approach for classifying local image patches. Lesion annotations were only needed during initial training, and the entire image classifier was trained on the CBIS-DDSM database and transferred to INbreast FFDM images using only a few samples for fine-tuning without relying on additional lesion annotations. Two steps were used to train a whole image classifier using VGG-16 and ResNet-50 models. First, a patch classifier was trained. From the patch classifier, a whole-image classifier was developed. A probabilistic grid of outputs was generated based on the recognition of local patches on images, and different results were obtained depending on different patch sets. The highest result achieved in this study was an area under the curve (AUC) of 0.95 using the independent test set from the INbreast database.

Tan et al. [59] proposed a convolutional neural network to detect breast cancer using mammograms. In this study, the mini-Mammographic Image Analysis Society (mini-MIAS) database was used, with 322 mammograms classified into normal, benign and malignant from the original database. These images were preprocessed, and only the abnormal tissue was cropped to extract only relevant information. This network was composed of a patch of 48 × 48 pixels as inputs, a convolution layer with a kernel size of 5 × 5, a pooling layer with a 2 × 2 filter and strides of 2, a learning rate of 0.003, and a training step of 20,000. With their proposed system, the highest accuracy achieved was 82.71%.

**Figure 8:**
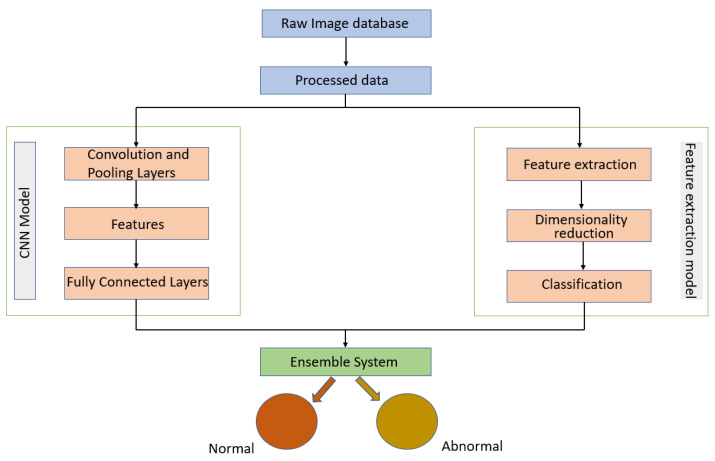
Diagram of the ensemble approach with a CNN and feature extraction for breast cancer detection proposed in [60].

Melekoodappattu et al. [60] used an integration method to develop a system for detecting cancer. Their method consisted of an ensemble approach developed to diagnose malignancies in breast tissue. A CNN model and feature extraction were the central components of the ensemble model, which can enhance classification efficiency. The images were preprocessed by using methods such as the median filter to remove noise and image enhancement to improve contrast. The extraction-based method relied on defining texture features and reducing their dimensions using uniform manifold approximation and projection. Several classifiers were used in conjunction with a nine-layer CNN model to diagnose cancer on the MIAS and DDSM repositories. Using the ensemble of a CNN and feature extraction, the accuracies achieved were 98.00% using the MIAS repository and 97.90% using the DDSM repository. As illustrated in Figure 8, their approach comprised four stages: pre-processing, CNN classification, feature extraction-based classification and an integration system.

Using deep convolutional neural networks (CNN), Khan et al. [7] proposed a method for classifying and segmenting breast abnormalities, such as calcifications, masses, asymmetry and carcinomas. The images have been subjected to various filtering techniques in order to select the most appropriate one, including Wiener filters to reduce image noise, inverse filtering to recover blurred images and median filters to reduce the amount of intensity variation between pixels while maintaining the sharpness of image edges [61]. They applied transfer learning by using a pretrained ResNet-50 model to the customized dataset created from the CBIS-DDSM and UPMC datasets, where UPMC comprised tomosynthesis images containing asymmetric breast abnormalities and mass images. Following that, data augmentation was applied to produce a selection of images based on a variety of datasets. To optimize the model further, an enhanced CNN was developed to adjust the learning rate. The results showed an accuracy of 88.00% in the classification of the abnormalities.

In [62], a convolutional neural network (CNN) architecture was proposed by Altan et al. to classify mammograms into 2 classes, normal and cancerous. The contribution was based on the observation that pruned CNN architectures can be used with a feature-based learning process to classify mammograms. The best CNN model consisted of 18 layers and achieved 92.84% accuracy, 95.30% sensitivity and 96.72% specificity.

Varela et al. [63] proposed a system to detect malignant masses on mammograms by applying an IRIS filter to segment suspicious regions. The database was selected from patient files at hospitals of the health district of Santiago de Compostela (Spain). After processing the mammograms, an adaptive threshold was applied to select and segment potential lesions. A total of five types of features were extracted from the segmented areas to distinguish between mass and false-positive (FP) detection. These images were characterized based on the output of the IRIS filter, gray levels, texture, contour-related and morphological features extracted from the images. Their IRIS filter was implemented using a region of support with a circular ring having an inner radius of 2.5 mm and an outer radius of 17.5 mm. A backpropagation neural network classifier was trained with a combination of seven features to reduce the number of false positives. The free-response receiver operating characteristic analysis was used to evaluate the performance of their system in a complete independent test set. On the basis of lesion evaluation, a sensitivity of 88.00% was achieved at an approximate FP rate per image of 1 while a sensitivity of 94.00% was achieved at 1.02 FP findings per image.

Using a variety of models, Salama et al. [64] proposed a new framework for breast cancer image segmentation and classification. A modified U-Net model was used for segmenting breast areas of 3 different databases, MIAS, DDSM and CBIS-DDSM. Furthermore, a number of models, including Inception-V3, DenseNet-121, ResNet-50, VGG-16 and MobileNet-V2, were employed to classify the segmented MIAS, DDSM and CBIS-DDSM databases as benign or malignant. Two different views, craniocaudal (CC) and mediolateral oblique (MLO), were employed to improve the system performance. The best results were achieved by using the Inception-V3 model on the DDSM dataset. It achieved 98.87% accuracy, 0.98 area under the curve (AUC), and 98.98% sensitivity. Combining MLO and CC views in the proposed framework was more efficient than using the MLO view alone.

Altameem et al. [65] developed an ensemble approach based on a Gompertz function by building fuzzy rankings of the deep CNN techniques Inception-V4, ResNet-164, VGG-11, and DenseNet121. This study employed four mammography datasets, including the Breast Cancer Digital Repository (BCDR), Mini Mammographic Image Analysis Society (Mini-MIAS), INbreast, and the Digital Database for Mammography Screening (DDSM), each with 1145 normal, benign and malignant images. As a result of the proposed approach, the suggested Inception-V4 ensemble model had an accuracy of 99.32%.

### 4.2. Thermography

Thermography is a type of breast cancer screening, also known as infrared imaging, where an infrared camera is used to detect heat patterns within the body and to measure blood flow [66]. It measures the temperature of a patient’s breast skin.

Pramanik et al. [67] proposed a method for segmenting breast tissue based on a series of three steps: background removal, inframammary fold detection and axilla detection. Otsu’s thresholding and gray level reconstruction techniques were used to remove the background of the image. Feature extraction was performed using multi-resolution analysis on breast region, and these features were then sent to a feed-forward neural network-based classifier to detect healthy and unhealthy breast tissue. In their proposed system, the results showed an accuracy of 90.48%, sensitivity of 87.60% and specificity of 89.73%.

Torres-Galván et al. [68] evaluated deep neural networks using automated techniques for breast thermogram classification. Seven deep learning architectures, AlexNet, GoogLeNet, ResNet-50, ResNet-101, InceptionV3, VGG-16 and VGG-19, were used to train 173 images. The best accuracy achieved was 91.18%, with a sensitivity of 100.00% and specificity of 82.35% with a VGG-16 convolutional neural network. Despite false positives, the results demonstrated the usefulness of deep neural networks for breast cancer pre-screening.

**Figure 9:**
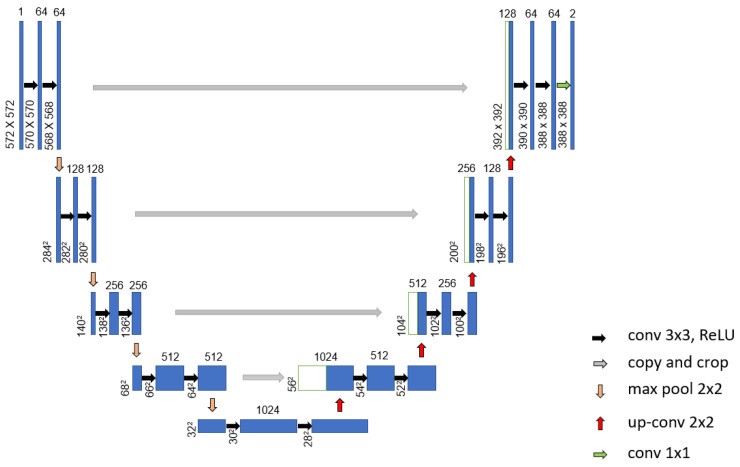
Architecture of the proposed U-Net network used in [70].

**Figure 10:**
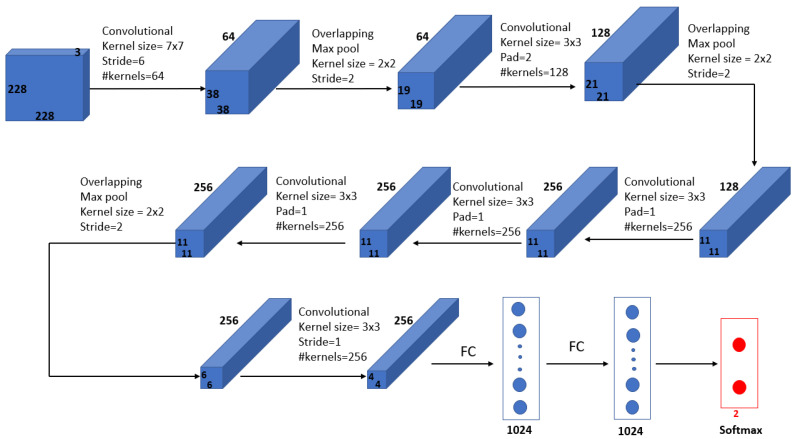
Architecture of the proposed CNN model in [69].

Mohamed et al. [69] proposed an automatic segmentation method for detecting breast cancer. The DMR-Database for Mastology Research-Visual Lab was used to evaluate the proposed method. The U-Net network, shown in Figure 9, was used to extract and isolate the breast regions from other areas of the body. Furthermore, a two-class CNN model was trained from scratch to classify the thermal images into normal and abnormal breast tissues. Using 1000 thermal images, the proposed system achieved an accuracy, sensitivity and specificity of 99.33%, 100.00% and 98.67%, respectively. Figure 10 illustrates the architecture of their proposed CNN model.

In [71], several convolutional neural network (CNN) models, such as ResNet-101, DenseNet, MobileNet-V2 and ShuffleNet-V2, were used for breast cancer detection. Static and dynamic DMR databases consisting of two classes, cancerous and healthy, were used for training. As a result, DenseNet classified all the databases correctly. In the case of ResNet-101 and MobileNet-V2, static datasets were correctly classified, whereas dynamic datasets obtained an accuracy of 99.60%. Only a 98.00% accuracy was achieved by ShuffleNet-V2.

Mahoro et al. [72] proposed a breast cancer detection system based on the segmentation and classification of breast thermograms. A vision-based Transformer called TransUNet was used to segment the breast region of interest. Each of four models, EfficientNet-B7, ResNet-50, VGG-16, and DenseNet-201, was evaluated on its performance in classifying segmented thermograms into healthy, sick, and unknown types. A total of 3989 breast thermograms from the DMR-Database for Mastology Research-Visual Lab was used. The results obtained using the ResNet-50 model included an accuracy and sensitivity of 97.26% and 97.26%, respectively.

Mishra et al. [73] used a deep convolutional neural network model to predict breast cancer. The proposed network consisted of five groups of convolutional, batch normalization, and rectified linear activation function (ReLU) layers and three max-pooling layers, followed by one dropout layer, one fully connected layer, one softmax layer and one classification layer. In this method, 680 thermal images were converted to grayscale, preprocessed, segmented and classified. As a result, the accuracy achieved was 95.80%, while sensitivity and specificity were 99.50% and 76.30%, respectively.

In [74], Ekici et al. suggested a new algorithm for feature extraction based on bio-data, image analysis and image statistics. Five processes were employed in the proposed method, namely data acquisition, image processing, segmentation, feature extraction, and classification. Breast segmentation was done using projection profile analysis. The segmented images were classified as normal or suspected by using a convolutional neural network (CNN) optimized by the Bayesian algorithm. The proposed network consisted of multiple layers, including convolutional, max-pooling, and fully connected layers, engaging multiple stages. Based on their proposed algorithm, which was implemented on 3895 thermal images, they obtained an accuracy of 98.95%.

### 4.3. Ultrasonography

A form of breast cancer screening known as ultrasonography is an alternative method for detecting breast cancer when mammography cannot be used [75]. It is useful when there is no way to see through the tissues or when the patient is pregnant or under the age of 25.

Becker et al. [76] tested a generic deep learning software (DLS) called ViDi Suite Version 2.0 against human readers for breast cancer diagnosis. The software identified and categorized anomalies in image data using deep learning algorithms. The breast ultrasound dataset used consisted of 632 images, 82 of which were malignant lesions and 550 of which were benign lesions. As for human interpretation, the training images were given to a 4th-year medical student and the validation images were presented to two radiologists under the assumption that, similar to the software, the student would learn solely from the images. A comparison between the performance of the DLS and human readers was conducted. Compared to human readers, the DLS took less time to evaluate the dataset, and its area under the curve (AUC) was 0.84.

Qian et al. [77] proposed an ensemble deep learning system for the classification of breast ultrasound images. A comparison with different models, including ResNet-18, VGG19, ResNet-50 and Inception-v3, all of which were embedded with the SENet block, was performed to identify the most effective base model for breast cancer prediction. The proposed system was trained on 10,815 breast ultrasound images derived from 634 patient cases involving 721 lesions. The ResNet-18 model with the SENet backbone provided better performance, and the results obtained for the area under the receiver operating curve (AUC) was 0.95 for multimodal images compared to 0.92 for bimodal images.

Almajalid et al. [78] developed a breast ultrasound image segmentation framework based on U-Net deep learning architecture. In order to improve the quality of images, they applied preprocessing techniques to a database of 221 images. With the help of the speckle-reducing anisotropic diffusion (SRAD) method, they reduced speckle noise while preserving the image features and also used histogram equalization to increase the contrast. Two-fold cross-validation was used to train and test the proposed model. As a result, the dice coefficient was 0.82, and the similarity rate was 0.69. As a final step, the segmentation result was post-processed to remove noisy regions.

Han et al. [79] proposed a GoogLeNet convolutionary neural network to identify distinct types of lesions and nodules on breast ultrasound images. A total of 7408 images of the breast were included in the dataset, of which 4254 were benign and 3154 were malignant. Histogram equalization, image cropping and margin augmentation were used in the proposed method. The optimal parameters were determined through ten-fold cross-validation with the training data. As result, they found an accuracy of about 90.00%, a sensitivity of 86.00%, and a specificity of 96.00%. With this method, radiologists can differentiate malignant lesions more accurately and in a shorter period of time.

Yap et al. [80] investigated the performance of three deep learning methods, a patch-based LeNet, a U-Net, and the transfer learning approach with a pretrained FCN-AlexNet, for the detection of lesions in breast ultrasounds. Two conventional datasets named Dataset A and Dataset B, having 306 and 163 images, respectively, were used. According to the results, the transfer learning FCN-AlexNet method performed better than the other two approaches with respect to the true-positive fraction (TFP), false positives per image (FPs/image) and F-measure on two datasets. With the transfer learning FCN-AlexNet model, they obtained a TPF of 0.98, FPs/image of 0.16, and an F-measure of 0.91 for Dataset A and a TPF of 0.92, FPs/image of 0.17, and an F-measure of 0.89 for Dataset B.

**Figure 11:**
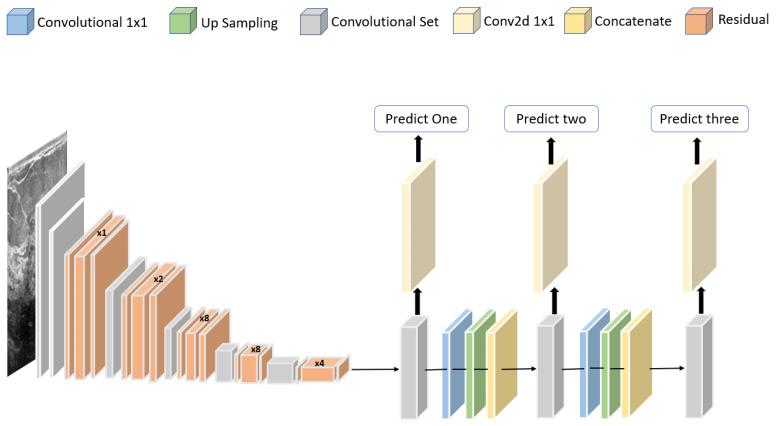
Structure of the modified DarkNet-53 deep model proposed in [81].

Jabeen et al. [81] proposed a framework for classifying breast cancer based on ultrasound images. A pretrained deep model, DarkNet-53, was modified and trained on augmented images. A new probability-based serial approach was used to fuse the best features chosen using reformed differential evolution and reformed gray wolf for classification based on machine learning algorithms. Using 780 images, the best accuracy obtained was 99.10%. The structure of the modified DarkNet-53 deep model is shown in Figure 11.

Based on a convolutional neural network, Tanaka et al. [82] developed a computer-aided diagnosis system for detecting malignant and benign masses in the breast using ultrasonography. By generating heat maps, they indicated the regions of interest used by a CNN for classification. The dataset contained 1536 images, of which 897 were malignant and 639 were benign. To fine-tune the ensemble network based on balanced training data, they combined two CNN models: VGG-19 and ResNet-152. The results showed a sensitivity of 90.90%, specificity of 87.00% and an area under the curve (AUC) of 0.95.

Ademola et al. [83] proposed a VEU-Net method consisting of the variant enhanced (VE) block and the concatenated convolutions for segmentation. A total of two datasets, containing, respectively, 264 and 830 breast ultrasound images, were used in combination with three different methods: the Dice measure, the Jaccard measure and the Hausdroff distance. Contrast-limited adaptive histogram equalization was applied to these images to improve the low quality of images and reduce noise using the bilateral filter. Afterwards, the preprocessed images were encoded using the VE block, and the concatenated convolutions were used to generate the segmentation mask. Based on the results, the proposed method achieved a high Dice measure for malignant images of 89.73% and for benign images of 89.62%.

Huang et al. [84] proposed an automatic segmentation algorithm based on a fuzzy fully convolutional network (FCN) and accurate fine-tuning post-processing. Contrast enhancement was applied to the image before wavelet features were used for image augmentation. Breast anatomy layers were mapped to improve the performance. With 325 images, the proposed system achieved a true-positive rate of 90.33%, a false-positive rate of 9.00%, an intersection over union rate of 81.29% on the tumor category and an overall intersection over union rate of 80.47% across the five categories: fat layer, mammary layer, muscle layer, background, and tumor.

Hijab et al. [85] proposed a deep learning technique for classifying breast ultrasound images into benign and malignant cases. Three approaches were used in the development of the proposed model: a training from scratch approach, a transfer-learning approach based on a pretrained VGG-16 CNN architecture and a fine-tuning approach. The dataset used consisted of 1000 images in the training set and 300 images in the test set. The dataset was augmented to overcome overfitting, and fine-tuning was applied along with the bottleneck features of the VGG16 pretrained model to increase the accuracy. An accuracy of 97.00% and an AUC of 0.98 were achieved by using the fine-tuned approach with a pretrained VGG-16.

Kim et al. [86] developed a weakly supervised deep learning algorithm that diagnosed breast cancer on ultrasound images without the image annotation. The DL algorithms were implemented using three networks: VGG-16, ResNet-34 and GoogLeNet. A comparison between manual and automatic ROI annotation with a fully supervised algorithm was made. The class activation maps of the breast masses were used to determine the accuracy of their localization using weakly supervised DL algorithms. The weakly supervised DL algorithms achieved excellent diagnostic performances in internal validation sets, with AUC values of 0.92–0.96 and in external validation sets, with AUC values of 0.86–0.90. All weakly-supervised algorithms, except for ResNet-34 (98.00%), detected malignant masses with 100.00% accuracy on both the internal and external validation sets.

Podda et al. [86] developed a convolutional deep autoencoder model for segmentation and radiomic extraction. In parallel with segmenting the breast lesions, radiomic features are extracted. In this study, 780 ultrasound images were used to train the models, including 437 benign images, 210 malignant images, and 133 normal images. In total, 354 conventional radiomics were extracted using a radiomic library, and its dimensionality decreased more than 29 times to 12 imaging biomarkers by applying the spectral mapping algorithm. A random forest model was trained, tuned, cross-validated, and tested to classify segmented lesions into malignant and benign types. As result, they obtained 78.50% accuracy for a maximum cross-validated model for a combination of radiomic groups.

### 4.4. Magnetic Resonance Imaging (MRI)

Magnetic resonance imaging (MRI) makes detailed pictures of the inside of the breast using radio waves and powerful magnets [87]. In most cases, it is used to measure the size of the cancer and detect other tumors in the breast.

Verburg et al. [88] proposed an automated triaging model based on 4581 breast MRI examinations by eliminating most examinations without lesions while identifying malignant lesions. In addition to discarding breasts with normal phenotypical variations, the model was trained to triage lesions based on eight-fold internal-external validation. Receiver operating characteristic analysis was employed to assess performance. When the proposed model was at 100.00% sensitivity for malignant lesions, 90.70% of the MRI exams with lesions were labeled as abnormal and directed to radiology review. As a result, 39.70% of MRI exams without lesions were dismissed by the proposed model. When comparing MRI examinations with and without lesions, the proposed model had an average AUC of 0.83.

Yunan et al. [89] developed a fusion CNN model based on dynamic contrast-enhanced MRI images to improve breast cancer diagnosis. The proposed network consisted of two branches, a deep branch with a composite grayscale tumor ROI image, and a shallow branch with seven analytical features as input. Among the 130 patients, 71 had malignant tumors and 59 had benign tumors. Three types of evidence criteria were used to interpret the CNN classification outcome: prediction probability, feature visualization and contributing dynamic scan time points. In a five-fold evaluation process, the proposed method achieved an accuracy of 87.70%, a precision of 91.20%, a sensitivity of 86.10% and an AUC of 0.91.

With the aim of improving breast MRI lesion classification specificity, Liu et al. [90] presented a weakly supervised deep learning approach without pixel-level segmentation. The proposed approach was based on the ResNet-101 architecture. In total, the dataset contained 288,685 image slices from 438 patients. Rather than just evaluating the region of interest (ROI) of the MRI image, their network evaluated the entire slice of the image to increase its specificity. In contrast to the process of manually defining ROI boundaries, the proposed approach reduced errors due to subjectivity. Using the proposed method, breast MRI images were classified into malignant and benign and achieved an AUC of 0.92, an accuracy of 94.20%, a sensitivity of 74.40% and a specificity of 95.30%.

**Figure 12:**
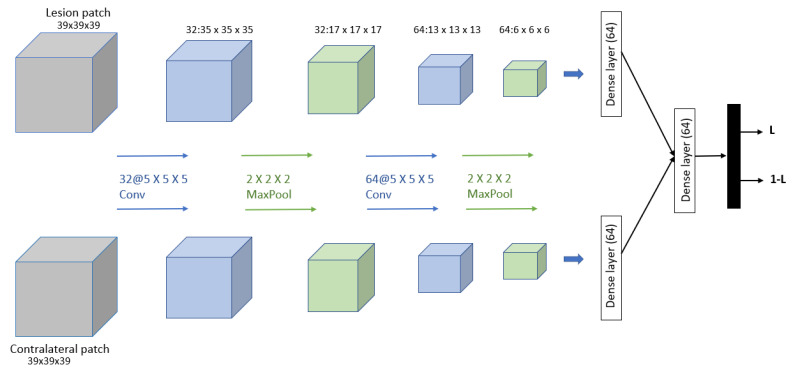
Architecture of the proposed CNN model in [91].

Dalmış et al. [91] developed a computer-aided detection (CADe) system based on the spatial information from early-phase MRI scans. Dynamic breast MRI protocol is used in this system, comprising precontrast and registered postcontract images. The process involved three steps: breast segmentation using U-Net architecture, candidate detection, and candidate classification using a 3D CNN with two patches as input and one convolutional, one max-pooling, and one dense layer for each input. The dataset consisted of 385 MRI scans, of which 161 are malignant lesions and 224 are benign lesions. As result, the proposed system obtained a significantly higher average sensitivity of 64.29%. The proposed 3D CNN is shown in Figure 12.

Using multiparametric magnetic resonance imaging, Hu et al. [92] developed a computer-aided diagnosis method based on deep transfer learning. This dataset contained MRI images of 927 unique lesions on 616 women. Dynamic contrast-enhanced (DCE) and T2-weighted MRI sequences were both used in this study, and feature extraction was performed using a pretrained convolutional neural network (CNN). The image fusion, feature fusion and classifier fusion were used, and the dataset was classified into benign or malignant using support-vector machine (SVM) classifiers. With the single-sequence classifiers, the AUC of DCE was 0.85, and the AUC of T2w was 0.78, while the AUCs of the multiparametric schemes were 0.85 for ImageFusion, 0.87 for FeatureFusion and 0.86 for ClassifierFusion. The best result obtained was with the feature fusion method.

Herent et al. [93] developed a deep learning model with the ability to detect and characterize lesions simultaneously. Using a single two-dimensional T1-weighted fat-suppressed MRI image, they developed a lesion characterization model. A ResNet50 Neural Network was used to extract features from images, and those images were then processed by the algorithm’s attention block that learned to detect anomalies in the images. They also fed the images into a second branch that averaged features over the selected areas. The features were then fitted to a logistic regression, which generated the output. A total of 335 images representing 17 histological subtypes of breast lesions were included in the dataset and grouped into 4 categories: mammary glands, benign lesions, invasive ductal carcinoma and other malignant lesions. Using the test set, the proposed model obtained a weighted mean AUC of 0.81.

Benjelloun et al. [94] proposed a deep learning approach for segmenting breast tumors on dynamic contrast-enhanced MRI (DCE-MRI) imaging data. The architecture was based on the U-Net fully convolutional neural network. A total of 86 DCE-MRIs from 43 adult patients with local breast cancer were acquired before and after chemotherapy. Annotations were performed manually in each breast tumor area to create ground-truth data. In total, 5452 slices were gathered from the DCE-MRIs for training and validation. Each breast slice was detected and segmented by the trained model. A mean intersection over union (IoU) of 76.14% was achieved by the proposed model.

Zhang et al. [95] proposed a method for breast segmentation using the U-Net architecture to quantify fibroglandular tissue volume in breast MRI. The segmentation was performed on the precontrast T1-weighted images without fat suppression. MRI images of 286 patients were used as a training set and were segmented based on their contralateral normal breasts. A template-based segmentation method was used to obtain the ground truth for breast and fibroglandular tissue. The 10-fold cross-validation algorithm was used to develop the final model. This resulted in a mean accuracy of 94.00% for breast tissue and 93.00% for fibroglandular tissue.

Using MRI images, Yurttakal et al. [96] proposed a method based on a convolutional neural network to classify lesions as malignant or benign. A multi-layer CNN architecture with online data augmentation was designed with only pixel information. Six groups of convolutional, batch normalization, rectified linear activation function (ReLU) layers and five max-pooling layers were present in the proposed network, followed by one dropout layer, one fully connected layer, and one softmax layer. Among the 200 tumorous regions in the breast MRI dataset, there were 98 that were benign and 102 that were malignant. The proposed method achieved an accuracy of 98.33%, a sensitivity of 100.00% and a specificity of 96.88%.

Amit et al. [97] proposed a new way of representing dynamic contrast-enhanced breast MRI lesions. Two different deep learning approaches were used to classify breast MRI lesions automatically. A designated convolutional neural network (CNN) as well as a pretrained VGG network were used to distinguish benign from malignant lesions. The proposed CNN consisted of three consecutive blocks of convolution, pooling, and rectified linear unit layers, followed by a fully connected layer and a softmax loss layer. In total, 123 female patients’ breast MRI exams were analyzed, consisting of 173 annotated lesions and resulting in 891 malignant images and 365 benign images. Comparing the proposed network to the pretrained model, the proposed network provided a higher level of classification accuracy of 83% accuracy with an AUC of 0.91.

Table 3 shows the reviewed work on breast cancer detection using deep learning.

## 5. Discussion

In this section, we will discuss the studies and challenges in the area of breast cancer research.

The use of deep learning in medical research has proven to be very effective in medical image analysis. Despite this, the available breast cancer datasets are not balanced, which can lead to inaccurate diagnosis. It is important to note that some informative datasets are not publicly available or are not annotated, which results in a lack of information for medical research. The consequences of misreading a medical imaging report can be severe. The study in [98] explained the fact that the specificity of artificial intelligence (AI) systems cannot completely replace an expert in radiology. However, there should be collaboration between radiologists and computer vision experts. In order to increase the accuracy of medical reports, research should focus on practical applications.

Our review explores the use of deep learning models to detect breast cancer with four different screening methods. With each screening method, we observe different databases have been made available for research and have been used to train the models. The three most common deep learning tasks in the breast cancer research field are image classification, object detection, and image segmentation. In order to obtain an efficient result, several models were implemented by considering learning attributes, weights and activations. Table 4 describes the best models and their results.

As shown in Table 4, Mohamed et al. [69] achieved the highest level of accuracy of 99.33% by segmenting 1000 thermal images using the U-Net network and then classifying them into normal and abnormal breast tissues using a pretrained CNN. In second place, Jabeen et al. [81] achieved an accuracy of 99.10% by classifying breast cancer from 780 ultrasound images using the pretrained deep model DarkNet-53. In third place, an accuracy of 98.95% was obtained by Ekici et al. [74] by classifying the 3895 thermal images as normal or suspected using a CNN optimized by the Bayesian algorithm. This was followed by an accuracy of 98.87% achieved by Salama et al. [64], who used a modified U-Net model to segment breast areas and Inception-V3 to classify the segmented DDSM database into benign or malignant categories. Lastly, an accuracy of 98.33% was achieved by Yurttakal et al. [96] using a multi-layer CNN architecture to classify 200 MRI images as malignant or benign.

Overall, we can see that the best-performing models used architectures based on segmenting the region of interest of the images and then classifying them. As the regions of interest are extracted and isolated, the models are able to focus on that area during training. Due to the fact that the models were tested on different datasets with varying preprocessing and enhancement, it is not feasible to perform a direct comparison of the different models. However, the analysis highlights important aspects of designing a suitable deep learning architecture for detecting breast cancer.

## 6. Conclusions

In addition to being the most common and most harmful disease, breast cancer detection presents a challenging issue. With the help of the AI tool, many applications were able to detect breast cancer even if there was no obvious tumor visible to the human eye. Several deep learning applications and techniques have been developed to assist radiologists in diagnosing medical images. Mammography, thermography, ultrasound and MRI are some of the screening approaches used to detect breast cancer at an early stage. By using different databases with different screening methods, researchers were able to train the models and improve the early detection of breast cancer in many ways.

However, there are still challenges to overcome, and more applications are needed to improve the accuracy of breast cancer detection. One of the challenges is the lack of balance in the available breast cancer datasets. Medical research can be constrained by the lack of information from some informative datasets that are not publicly accessible or unannotated. In order to improve accuracy, recent deep learning techniques such as Transformers can be used, as they have demonstrated remarkable improvements in image classification, object detection, and segmentation. In future work, a new computer-aided system based on such techniques may be useful. For image classification, we can use the Vision Transformer (ViT) model [99], a Transformer encoder model that can classify breast images as malignant or benign. The detr-resnet-50 model [100], an encoder-decoder transformer with a ResNet-50 backbone that can help detect breast cancer. Segmentation techniques such as TrSeg [101], a Transformer for semantic segmentation, can also be used to extract region of interest for breast cancer. Even though deep learning is effective in analyzing medical images, there are not enough balanced breast cancer datasets, which can lead to inaccurate diagnosis. Increasing medical report accuracy requires access to some useful datasets, informative review and a focus on practical applications. This review provides an overview of the most recent research on integrating deep learning into medical imaging for breast cancer diagnosis, as well as some techniques that may be useful in guiding future research in the field.

## Figures and Tables

**Table 1:** Metrics used in deep learning for breast cancer detection. TP = true positive, TN = true negative, FP = false positive and FN = false negative.

Methodology	Metric	Definition	Note
Classification	Accuracy	Acc = (TP + TN)/(TP + TN + FP + FN)	The number of correctly classified samples
	Sensitivity	Recall = (TP)/(TP + FN)	True-positive rate (TPR) or recall
	Specificity	Specificity = (TP + TN)/(TP + TN + FP + FN)	True-negative rate (TNR)
	Precision	Precision = (TP)/(TP + FP)	The ratio between correctly classified positive samples and the total number of samples classified as positive
	F1-Score	F1-Score = 2 ∗ (Precision ∗ Recall)/(Precision + Recall)	The classification ability of the classifiers
	ROC	The performance of a classification model at all classification threshold illustrated as a graph	Receiver operating characteristic curve
	AUC	Used to summarize the ROC curve and measure how well a classifier can distinguish between classes	Area under the ROC curve
	PR curve	Shows the precision/recall trade-off for different probabilities	Precision-recall curve
	Average precision	$\mathrm{AP}=\int_{0}^{1}p\left(r\right)dr$	Identifying the area under the precision-recall curve
Object detection	mAP	$\mathrm{mAP}=\frac{1}{N}\sum_{i=1}^{N}{\mathrm{AP}}_{i}$	Mean average precision
	IoU	IoU = Area of Overlap/ Area of union	Intersection over union
	NMS	The selection of the best bounding box for mAP evaluation over an object	Non-maximum suppression
Segmentation	PA	Indicate the percentage of pixels correctly classified in the image	Pixel accuracy
	mPA	Computes the mean of pixel accuracy	Mean pixel accuracy
	MIoU	Computes the mean of IoU	Mean intersection over union

**Table 2:** Various public databases and their characteristics.

Dataset	Modality	Year	No. of Images	Resolution	Image Type
MIAS [42]	Mammography	1994	322	8 bits/pixel	PGM
Magic-5 [43]	Mammography	1999	3369	16 bits/pixel	DICOM
BancoWeb [45]	Mammography	2010	1473	12 bits/pixel	TIFF
INbreast [44]	Mammography	2012	410	14 bits/pixel	DICOM
DDSM [46]	Mammography	1999	10,480	8 or 16 bits/pixel	LJPEG
CBIS-DDSM [47]	Mammography	2017	3468	16 bits/pixel	DICOM
OPTIMAM [48]	Mammography	2010–present	2,889,312	-	DICOM
BCDR [49]	Mammography	2009–present	3703	14 bits/pixel	TIFF
DMR-Database [24]	Thermography	2010–present	5760	-	JPG
BUS [50]	Ultrasound	2017	250	-	BMP
Breast Ultrasound Dataset [25]	Ultrasound	2018	780	-	PNG
Duke-Breast-Cancer-MRI [55]	MRI	2000–2014	922	-	DICOM
RIDER Breast MRI [56]	MRI	2006	1500	-	DICOM

**Table 3:** Some of the state-of-the-art methods using breast cancer images. Acc: accuracy, Sn: sensitivity, AUC: area under the curve, Dc: Dice coefficient, IoU: intersection over union and AUC-ff: AUCs of the feature fusion.

Modality	Ref.	Dataset	Methodology	Architecture	Results
Mammography	[58]	INbreast	Classification	VGG-16 + ResNet-50	AUC = 0.95
	[60]	MIAS	Detection	Nine-layer CNN and feature extraction	Acc = 98.30%
	[64]	DDSM	Segmentation and classification	U-Net and Inception-V3	Acc = 98.87%
	[7]	CBIS-DDSM + UPMC	Segmentation and classification	ResNet-50	Acc = 88.00%
	[62]	DDSM	Classification	Eighteen-layer CNN	Acc = 92.84%
	[63]	386 images	Segmentation	IRIS filter and adaptive threshold	Sn = 94.00%
	[65]	BCDR + DDSM + INbreast + Mini-MIAS	Classification	Fuzzy Ensemble Modeling Techniques	Acc = 99.32%
Thermography	[68]	173 images	Classification	VGG-16	Acc = 91.18%
	[69]	1000 images	Segmentation and classification	U-Net and pretrained CNN	Acc = 99.33%
	[72]	3989 images	Segmentation and classification	TransUNet + ResNet-50	Acc = 97.26%
	[74]	3895 images	Classification	CNN optimized by the Bayesian algorithm	Acc = 98.95%
	[73]	680 images	Classification	Deep CNN	Acc = 95.80%
Ultrasound	[76]	632 images	Classification	Generic deep learning software (DLS)	AUC = 0.84
	[77]	10,815 images	Classification	Multimodal deep learning model	AUC = 0.95
	[78]	221 images	Segmentation	U-Net	Dc = 0.82
	[79]	7408 images	Classification	GoogLeNet	Acc = 90.00%
	[82]	1536 images	Classification	VGG-19 + ResNet-152	AUC = 0.95
	[85]	1000 images	Classification	Pretrained VGG-16	Acc = 97.00%
	[81]	780 images	Classification	Pretrained DarkNet-53	Acc = 99.10%
MRI	[94]	86 images	Segmentation	U-Net	mean IoU = 76.14%
	[90]	288,685 images	Classification	ResNet-101	Acc = 94.20%
	[93]	335 images	Detection	Pretrained ResNet-50	mean AUC = 0.81
	[89]	130 images	Classification	Sixteen-layer CNN	Acc = 87.70%
	[95]	286 images	Segmentation	U-Net	Acc = 94.00%
	[92]	927 images	Feature extraction and computer-aided diagnosis method	Pretrained CNN and SVM classifier	AUC-ff = 0.87
	[96]	200 images	Classification	Multi-layer CNN	Acc = 98.33%
	[91]	385 images	Detection	3-D CNN	Sn = 0.64
	[97]	1256 images	Classification	Pretrained CNN	Acc = 83.00%

**Table 4:** Comparison of performance metrics of the best models. Acc: Accuracy.

Ref.	Modality	Dataset	Methodology	Model	Acc (%)
[69]	Thermography	1000 images	Segmentation and classification	U-Net and pretrained CNN	99.33
[81]	Ultrasound	780 images	Classification	Pretrained DarkNet-53	99.10
[74]	Thermography	3895 images	Classification	CNN optimized by the Bayesian algorithm	98.95
[64]	Mammography	DDSM	Segmentation and classification	U-Net and Inception-V3	98.87
[96]	MRI	200 images	Classification	Multi-layer CNN	98.33

## Data Availability

Not applicable.
